# Industry Efficiency and Total Factor Productivity Growth under Resources and Environmental Constraint in China

**DOI:** 10.1100/2012/310407

**Published:** 2012-12-27

**Authors:** Feng Tao, Ling Li, X. H. Xia

**Affiliations:** ^1^Institute of Industrial Economics, Jinan University, Guangzhou, Guangdong 510632, China; ^2^College of Resources and Environment, Guangdong University of Business Studies, Guangzhou, Guangdong 510320, China; ^3^Institute of China's Economic Reform & Development, Renmin University of China, Beijing, Haidian 100872, China

## Abstract

The growth of China's industry has been seriously depending on energy and environment. This paper attempts to apply the directional distance function and the Luenberger productivity index to measure the environmental efficiency, environmental total factor productivity, and its components at the level of subindustry in China over the period from 1999 to 2009 while considering energy consumption and emission of pollutants. This paper also empirically examines the determinants of efficiency and productivity change. The major findings are as follows. Firstly, the main sources of environmental inefficiency of China's industry are the inefficiency of gross industrial output value, the excessive energy consumption, and pollutant emissions. Secondly, the highest growth rate of environmental total factor productivity among the three industrial categories is manufacturing, followed by mining, and production and supply of electricity, gas, and water. Thirdly, foreign direct investment, capital-labor ratio, ownership structure, energy consumption structure, and environmental regulation have varying degrees of effects on the environmental efficiency and environmental total factor productivity.

## 1. Introduction

 Great achievements have been made in China's economy during the past three decades of reform and opening up. However, with rapid economic growth, the depletion of natural resources and the environmental degradation have become increasingly prominent. Based on a forecast for 2005–2035, China is to replace the USA as the world's leading embodied energy consumer in 2027, when its per capita energy consumption will be one quarter of that of the USA [1]. What's more, the total cost of environmental degradation and ecological damage reached about 2037 billion US dollars, accounting for 3.8% of the gross domestic product (GDP) in 2009 [2]. The problems of resources and environment not only have brought huge losses to China's economic and social development, but also may directly lead to unsustainable development in the future. Therefore, the 12th five-year plan of China request policy makers promote the coordination and sustainability of economic development. In addition, carbon emissions associated with industry transfer and international trades are illustrated in terms of impacts on global climate policies [3], so the globalization also promotes China to pay more emphasis on energy saving and environment protection.

 In much of the contemporary literature, researchers have been studying the changes in China's efficiency and productivity and their influence on economic growth and transformation from various perspectives. Nevertheless, with increasingly prominent problems of resources and environment in the process of economic development, a growing number of researchers believe that resources and environment are not only endogenous variables, but also rigid constraints on economic development [4, 5]. Therefore, when evaluating economic performance by total factor productivity (TFP), it is necessary to consider the resource and environmental factors which have tremendous impacts on economic development as well as a traditional factors such as capital and labor. In fact, resource and environmental factors have been added into efficiency and productivity analysis framework to reestimate China's economic growth efficiency and TFP in recent literature which draws many valuable conclusions [6–9].

 However, among these literatures, most of their data are based on subprovincial level in China, and very few of them are carried out from the subindustrial level in China. As Jorgenson and Stiroh [10] pointed out that economic growth is obviously different among sectors and industries, we should use subindustry data to describe the panorama of economic growth. In addition, due to less output potential loss for all the allocation alternatives, the sector regulation strategy is shown to be more effective than the province regulation strategy [11]. The rapid growth of China's industry highly depends on energy and environment, and industry plays the most important role in the energy saving and emission reduction of national economy. Therefore, it is significantly necessary to measure the costs of energy and environment. 

 Since traditional distance function cannot estimate the harmful effects of environmental pollution, many studies use indirect methods to calculate TFP with the consideration of pollutant emissions, which is obviously too simplified. Some researchers use radical and oriented data envelopment analysis (DEA) to compute directional distance function in order to simulate the harmful effects of environmental pollution, but this method will overestimate the efficiency of the evaluation object [12]. In contrast, nonradical and nonoriented directional distance function which is slack-based measure (SBM) and Luenberger productivity index can overcome the above deficiencies in the measurement of environmental efficiency and environmental TFP [13, 14]. 

 In addition, only one or several bad or undesirable outputs have been considered in the existing literature. However, for China's industry at this stage, all energy inputs and pollutant emissions should be taken into account, by which environmental efficiency and environmental TFP can reflect the quality contribution to economic growth more precisely [15].

 Therefore, on the basis of existing literature, this paper aims to use SBM directional distance function to measure environmental efficiency and its components of 36 subindustries of China's industry, use SBM directional distance function and the Luenberger productivity index to measure the environmental TFP and its components, then test, and compare the differences of the determinants' impacts.

## 2. Model Specification

 Different from the traditional production function, production technology considering energy and environment must reflect resource saving and environmental protection. Since resources can be introduced into productivity analysis framework as traditional inputs (such as capital and labor), the difficulty of constructing production frontier function is how to take environmental factors into account. In order to combine energy and environment factors with traditional economic factors (capital, labor, and output), according to Färe et al. [16], this paper considers every subindustry of China's industry as a decision making unit (DMU) and construct the optimal production frontier of each period. It is assumed that there are *N* kinds of inputs *x* = (*x*_1_,…, *x*_*N*_) ∈ *R*_*N*_^+^ in every subindustry, *M* kinds of good outputs *y* = (*y*_1_,…, *y*_*M*_) ∈ *R*_*M*_^+^, and *I* kinds of bad or undesirable outputs *b* = (*b*_1_,…, *b*_*I*_) ∈ *R*_*I*_^+^. Then, at the stage of *t* = (1, …, *T*), the subindustry of *k* = (1, …, *K*), the input and output vectors are (*x* ^*t*,*k*′^, *y* ^*t*,*k*′^, and *b* ^*t*,*k*′^), and the weight for each section of observation value is *λ*_*k*_^*t*^. Using data envelopment analysis (DEA), the environmental technology model is given by

(1) Pt(xt) ={(yt,bt):∑k=1Kλktykmt≥ykmt,∀m;∑k=1Kλktbkit=bkit,   ∀i;∑k=1Kλktxknt≤xknt,∀n;∑k=1Kλkt=1,λkt≥0,∀k}.

Because production frontier of every subindustry may lead to nonoptimal scale of production when considering imperfect competition and externality, a restriction is defined as ∑_*k*=1_^*K*^ *λ*_*k*_^*t*^ = 1, meaning that the production frontier reflects the hypothesis of variable returns to scale (VRS); If the restriction ∑_*k*=1_^*K*^ *λ*_*k*_^*t*^ = 1 is removed, then all firms can produce under the conditions of optimal scale, which means the production frontier reflects the hypothesis of constant returns to scale (CRS).

### 2.1. SBM Directional Distance Function

According to Fukuyama and Weber [14], SBM directional distance function considering resources and environment is defined as

(2) SVt→(xt,k′,yt,k′,bt,k′,gx,gy,gb)  =max⁡sx,sy,sb(1/N)∑n=1N(snx/gnx)2   +(1/(M+I))[∑m=1M(smy/gmy)+∑i=1I(sib/gib)]2    s.t.∑k=1Kλktxknt+snx=xk′nt, ∀n;       ∑k=1Kλktykmt−smy=yk′mt, ∀m;      ∑k=1Kλktbkit+sib=bk′it, ∀i;      ∑k=1Kλkt=1,λkt≥0,∀k;snx≥0, ∀n;      smy≥0, ∀m; sib≥0, ∀i,

where $\overset{\to }{S_{V}^{t}}$ denotes the directional distance function under VRS. If the weight variable and the constraint of 1 are removed, then $\overset{\to }{S_{c}^{t}}$ is a directional distance function under CRS; (*x* ^*t*,*k*′^, *y* ^*t*,*k*′^, and *b* ^*t*,*k*′^) refer to the input vector of each subindustry, good output vector, and bad or undesirable output vector; (*g* ^*x*^, *g* ^*y*^, and *g* ^*b*^) represent the direction vector of input compression, good output expansion, and bad or undesirable output compression; (*s*_*n*_^*x*^, *s*_*m*_^*y*^, and *s*_*i*_^*b*^) denote the slack variable of input, good output and bad or undesirable output; Slack variable measures observations' deviation from the production frontier, therefore (*s*_*n*_^*x*^, *s*_*m*_^*y*^, and *s*_*i*_^*b*^) indicates excessive use of inputs, underproduction of good outputs, and excessive emission of bad or undesirable outputs. Therefore, the target function is to maximize the sum of input-inefficiency average and output-inefficiency average. According to Cooper et al. [12], the above technical inefficiency can be decomposed as.

*Inputs inefficiency*:

(3) $$\begin{array}{c} IE_{x}=\frac{1}{2N}\sum_{n=1}^{N}\frac{s_{n}^{x}}{g_{n}^{x}}. \end{array}$$

*Good outputs inefficiency*:

(4) $$\begin{array}{c} IE_{y}=\frac{1}{2\left(M+L\right)}\sum_{m=1}^{M}\frac{s_{m}^{y}}{g_{m}^{y}}. \end{array}$$

*Bad or undesirable outputs inefficiency*:

(5) $$\begin{array}{c} IE_{b}=\frac{1}{2\left(M+L\right)}\sum_{l=1}^{L}\frac{s_{l}^{b}}{g_{l}^{b}}. \end{array}$$

### 2.2. Luenberger Productivity Index

 According to the existing literature, there are three main indexes to measure productivity: Malmquist index extended by Färe et al. [17], Luenberger productivity index developed by Chambers et al. [18], and Malmquist-Luenberger productivity index extended by Chung et al. [19]. Compared with Malmquist index and Malmquist-Luenberger productivity index, Luenberger productivity index does not need to choose the measuring orientation and make change in equal proportion. Therefore, Luenberger productivity index is more suitable for measuring the environmental efficiency and the environmental TFP which accounts for energy input and pollution emission.

 According to Chambers et al. [18], Luenberger productivity index between stage *t* and *t* + 1 is

(6) LTFPtt+1 =12{[SCt→(xt,yt,bt;g)−SCt→(xt+1,yt+1,bt+1;g)]    +[SCt+1→(xt,yt,bt;g)−SCt+1→(xt+1,yt+1,bt+1;g)]}.

Following Grosskopf [20], Luenberger productivity index can be further decomposed into pure efficiency change (LPEC), pure technical progress (LPTP), scale efficiency change (LSEC), and technical progress scale change (LTPSC)

(7) LTFP=LPEC+LPTP+LSEC+LTPSC,LPECtt+1=Svt→(xt,yt,bt;g)−Svt+1→(xt+1,yt+1,bt+1;g),LPTPtt+1 =12{[Svt+1→(xt,yt,bt;g)−Svt→(xt,yt,bt;g)]    +[Svt+1→(xt+1,yt+1,bt+1;g)−Svt→(xt+1,yt+1,bt+1;g)]},LSECtt+1 =[SCt→(xt,yt,bt;g)−SVt→(xt,yt,bt;g)]  −[SCt+1→(xt+1,yt+1,bt+1;g)−SVt+1→(xt+1,yt+1,bt+1;g)],LTPSCtt+1 =12{[(SCt+1→(xt,yt,bt;g)−SVt+1→(xt,yt,bt;g))     −(SCt→(xt,yt,bt;g)−SVt→(xt,yt,bt;g))]   +[(SCt+1→(xt+1,yt+1,bt+1;g)      −SVt+1→(xt+1,yt+1,bt+1;g))     −(SCt→(xt+1,yt+1,bt+1;g)       −SVt→(xt+1,yt+1,bt+1;g))]}.

When the above five values are greater than 0, they, respectively, indicate the productivity improvement, efficiency improvement, technical progress, scale efficiency improvement, and technical deviation CRS, conversely reverses. While it is necessary to use eight directional distance functions to decompose Luenberger productivity index, four of them belong to CRS hypothesis, and the other four are estimated under the condition of VRS hypothesis. 

## 3. Measurement of Environmental Efficiency and Environmental TFP

### 3.1. Outputs and Inputs

#### 3.1.1. Outputs

**Good Outputs**

industrial output is the most important good outputs, and it refers to gross industrial output value of 36 subindustries over the period from 1999 to 2009, which can be obtained from China Statistical Yearbook, published by National Bureau of Statistics of China (NBSC) [21]. The data should be transformed as 1990's constant price according to the producer price index (PPI) for manufactured goods.

**Undesirable Outputs**

considering the emissions of industrial pollutants, the bad or undesirable outputs should consist of industrial wastewater, carbon dioxide, sulfur dioxide, and solid waste. Emissions of wastewater, sulfur dioxide, and solid waste of each subindustry can be collected from NBSC. Unfortunately, there is no data of carbon dioxide emissions from NBSC, so this study follows Chen's methods [15] to use the reference approach in the Guidelines for National Greenhouse Gas Inventories provided by Intergovernmental Panel on Climate Change (IPCC) in 2006. Carbon dioxide emissions could be calculated as

(8) $$\begin{array}{c} C_{t}=\sum_{i=1}^{3}C_{i,t}=\sum_{i=1}^{3}E_{i,t}\times \text{NC}{\text{V}}_{i}\times \text{CE}{\text{F}}_{i}\times \text{CO}{\text{F}}_{i}\times \left(\frac{44}{12}\right). \end{array}$$

Here, the emissions of carbon dioxide are denoted by *C*, the types of primary energy (coal, oil, and natural gas) by *I* = (1, 2, 3), the consumption of energy by E. Meanwhile, NCV represents the average net calorific value of the primary energy obtained from China Energy Statistics Yearbook published by NBSC [22]. CEF represents the carbon emission factor provided by IPCC; COF represents the carbon oxidation factor; 44 and 12 correspond, respectively, to the molecular weight of carbon dioxide and carbon.

#### 3.1.2. Inputs

 Energy input should be considered as important as capital and labor. In the light of the majority of literature, this paper takes the number of employees every year as labor input and the energy consumption as energy input in subindustries, both of which can be inquired from NBSC [18]. Capital stock is one of the most important inputs, but NBSC does not provide details of the capital stock data; therefore, capital stock data need to be estimated. This paper estimates industry capital stock data with the method of perpetual inventory. Obviously, the calculation of capital stock of each year should be based on the capital stock of base year, depreciation rate, and constant price of investment. Following Chen's method [15], this study gets capital stock of 1980 as capital stock of the base year. The depreciation rates of subindustry are estimated with the data of depreciation value and fixed assets value from Chinese Statistical Yearbook and Chinese Industry Economy Statistical Yearbook published by NBSC [23]. This paper constructs the investment sequence data based on the difference of fixed assets and then converts them to constant price of 1990 by the investment price index of each year. After that, with the perpetual inventory method, capital stock data of various subindustries are calculated over the period from 1999 to 2009. 

### 3.2. Environmental Efficiency and Its Components

 Based on SBM directional distance function and Luenberger productivity index, this paper measures the environmental efficiency and environmental TFP by the software package Excel Solver Prem Platform V5.5 which is widely used in the present study. Environmental inefficiency values of every subindustry under the assumption of CRS and VRS are measured, respectively, and the results are given in Table 1.

 Since the environmental efficiency value under VRS assumption does not consider scale efficiency, the value under VRS assumption must be lower than CRS assumption, which is confirmed in Table 1. Under CRS assumption industrial production is in the conditions of optimal scale. However, many factors such as imperfect competition and externality may lead to nonoptimal scale. Therefore, when the value under CRS assumption is different from that under VRS assumption, the task is to analyze the efficiency under VRS assumption [24]. This study will focus on the analysis of environmental efficiency and its components under VRS assumption.

 The total value of environmental inefficiency of China's industry is 60.8%. The main source of environmental inefficiency is the inefficiency of gross industrial output value (14.7%), followed by the inefficiency of energy consumption (10.7%), capital stock (7.1%), SO_2_ (6.7%), solid waste (6.7%), CO_2_ (6.5%), and wastewater (5.7%), and the inefficiency value of employee (2.6%) is far lower than other outputs and inputs. Therefore, the keys of improvement of environmental efficiency are growth of industrial output, energy saving and reduction of pollutant emissions.

 In order to show the difference of environmental efficiency among subindustries due to industrial characteristics, this study classifies 36 two-digit code industries into three categories according to industrial classification standards provided by NBSC. The three industrial categories are mining, manufacturing, and production and supply of electricity, gas, and water. Table 1 shows that the highest environmental inefficiency value is mining (66.3%), followed by production and supply of electricity, gas, and water (59.6%) and manufacturing (56.5%). Inefficiency of gross industrial output value, energy consumption, and pollution emissions are the main sources of these three categories' environment inefficiency. The environmental inefficiency value of gross industrial output value of mining (19.5%) and production and supply of electricity, gas and water (16.6%) are much higher than manufacturing (7.9%). The inefficiency value of energy consumption of all three categories is more than 10%, which indicates that the task of energy saving of China's industry is very heavy.

### 3.3. Environmental TFP and Its Components

 The environmental TFP and its components are given in Table 2. The mean value of environmental TFP of China's industry over the period from 1999 to 2009 is 4.51%; in other words, the environmental efficiency of China's industry increased by 4.51% each year. This result is obviously lower than the traditional TFP without considering energy input and pollution outputs. About the components, the pure efficiency change is −3.69%, pure technical progress is 4.93%, scale efficiency change is 2.88%, and technical progress scale change is 0.39%. It means that technological innovation denoted by pure technical progress makes significant contributions to the improvement of the environmental TFP of China's industries. Therefore, technological innovation is the main driving factor of upgrading and sustainable development of China's industry. While the contribution of pure efficiency change is negative, and the contribution of technical progress scale change is small.

 The mean value of environmental TFP of mining is 5.46%, which is much higher than production and supply of electricity, gas, and water (2.49%), but lower than manufacturing (5.58%). Pure efficiency change and pure technical progress make the greatest contribution to the environmental TFP of mining; the mean values of which are 10.48% and 9.66%, respectively. 

 The main source of improvement of environmental TFP of manufacturing is pure technical progress and technical progress scale change; the mean values of which are 2.88% and 2.25%, respectively. Among the 28 subindustries of manufacturing, the value of environmental TFP of manufacture of communication equipment, computers and other electronic equipment (17.57%) is the highest, followed by manufacture of measuring instruments and machinery for cultural activity and office work (14.63%). Pure technical progress makes the greatest contribution to the former's environmental TFP, while scale efficiency change makes the greatest contribution to the latter's environmental TFP. The environmental TFP of some industries is very low, less than 2%, such as manufacture of leather, fur, feather and related products, manufacture of paper and paper products, processing of petroleum, coking, processing of nuclear fuel, and manufacture of chemical fibers, most of which are pollution-intensive industries.

 Because of the negative value of scale efficiency change and low value of pure efficiency change, the mean value of environmental TFP of production and supply of electricity, gas, and water is much lower than that of manufacturing and mining.

## 4. Determinants of Environmental Efficiency and Environmental TFP

### 4.1. Data

 What determines the environmental efficiency and environmental TFP of China's industry? Loko and Diouf fully analysed the determinants of productivity growth [25]. Based on the recent literature and context of China's economic transformation, the most important determinants of environmental efficiency and environmental TFP are as follows.

#### 4.1.1. Capital Structure (*X*_1_)

Capital structure is denoted by the proportion of value-added of foreign direct investment (FDI) enterprises in the added value of industrial enterprises above designated size. China has received significant FDI inflows for the past three decades, and FDI has been an important factor influencing industrial efficiency and productivity growth. Zhou et al. pointed out that domestic firms in industries which have more FDI or have a longer history of FDI tend to have lower productivity [26]. Estimating the influence of FDI on efficiency and TFP of China's industry under resources and environment constraint is actually a test of “pollution haven hypothesis” [27].

#### 4.1.2. Endowment Structure (*X*_2_)

 Endowment structure is denoted by capital-labor ratio. Capital and labor are sources of comparative advantage for most industries. The rising of capital-labor ratio means capital deepening which is an important determinant of industrial efficiency and productivity growth. Empirical studies show that the elasticity of substitution between capital and labor is larger than the one in developed countries but smaller than that in developing countries [28]. There are, however, several aspects of China's industrial strategy that have partially offset the trend toward capital deepening [29]. Therefore, this paper attempts to test the influence of capital deepening on efficiency and productivity under resources and environment constraint.

#### 4.1.3. Ownership Structure (*X*_3_)

 Ownership structure is denoted by the proportion of added value of state-owned enterprises (SOEs) covering the total added value of industrial enterprises above designated size. At the outset of the transition towards a market economy, the governments in developing countries envisioned that privatization would be an efficient way to improve performance and productivity. The reform of state-owned enterprises has greatly affected the profitability and productivity of Chinese industrial firms [30]. Incentive mechanism based on property rights may determine the environmental efficiency and environmental TFP through imperfect competition and pollution externality.

#### 4.1.4. Energy Consumption Structure (*X*_4_)

 Energy consumption structure is denoted by the proportion of electricity consumption accounted for total energy consumption. Different kinds of energy have different costs and pollution emissions which will influence the environment efficiency and environmental TFP.

#### 4.1.5. Intensity of Environmental Regulation (*X*_5_)

 China has adopted various policy measures to control industrial pollution. We need to assess the impact of pollution regulations on industrial productivity. Using the method of composite index, this paper builds a complex measurement system of China's industrial intensity of environmental regulation. This system has a target layer (intensity of environmental regulation) and three evaluation layers (waste water, waste gas, and solid waste).

 The main data sources are China Statistical Yearbook, China Energy Statistics Yearbook, Chinese Industry Economy Statistical Yearbook, and China Economic Census Yearbook published by NBSC [21–23, 31]. Some individual missing values are supplemented by linear interpolation. As the lower value of environmental inefficiency indicates higher environmental efficiency, in order to make the regression results consistent with the tradition, we use the formula *E* = 1/(1 + *IE*) to transform values of environmental inefficiency into values of environmental efficiency. Since the transformation value is between 0 and 1, we should choose the Tobit regression model. At the same time, because the environmental TFP should be analyzed dynamically and LTFP index is compared with last year, it is essential to transform the four types of index above into cumulative growth index taking 1999 as the base period. Because some values are negative, according to Managi and Jena [32], all values should be added one, and then the values through logarithmic transformation can be used as the dependent variable of the model.

### 4.2. Estimation Results

 The estimation results are given in Table 3. Hausman test shows that it's better to choose fixed effect model.

 Capital structure (*X*_1_) has a negative effect on both environmental efficiency and environmental TFP, that is to say, the increase of FDI reduced the industrial environmental efficiency and environmental TFP level; “pollution haven hypothesis” gets the verification here.

 The coefficients of capital labor ratio (*X*_2_) except the regression in CRS assumption are positive and significant, which indicates that capital deepening of China's industry can accelerate technological innovation and promote energy saving and emission reduction.

 The coefficients of ownership structure (*X*_3_) are not significant both for VRS hypothesis and the CRS hypothesis, but the ownership structure has significantly positive influence on environmental TFP. The estimation result indicates that the influence of ownership depends on complex factors and is not easy to be expected.

 The coefficients of energy consumption structure (*X*_4_) have significantly positive influence on environmental efficiency and environmental TFP. While the electricity consumption increased by 1%, the environmental efficiency and environmental TFP increased by more than 15%. The empirical results show that compared with coal, petroleum, and other fossil energy, electricity is clean energy, which can greatly improve China's efficiency and productivity under resource and environmental constraints.

 Environmental regulation intensity (*X*_5_) has a negative impact on the environmental efficiency under the hypotheses of VRS and CRS, but positive impact on the environmental TFP. This empirical result shows that environmental regulation will increase enterprise production costs and lead the environmental regulation and enterprise competitiveness to be in a dilemma in the short term.

## 5. Conclusions and Policy Implications 

 Using SBM directional distance function and Luenberger productivity index, this paper measures the environmental efficiency, environmental TFP, and its components at the level of subindustry in China over the period from 1999 to 2009 and tests the impacts of industrial capital structure, endowment structure, ownership structure, energy consumption structure, and intensity of environmental regulation. The findings of this study are crucial for environment administration and industrial upgrading. The specific policies are suggested as follows. 

### 5.1. Optimizing Industrial Structure

Considering the fact that both environmental efficiency and environmental TFP are different among subindustries, the government should accelerate the development of high-tech industries and environment friendly industries and limit the development of pollution-intensive industries and energy-intensive industries. The policy makers should also make vigorous guidance to draw FDI to high-tech industries and environmental-friendly industries and promote the industrial upgrading of FDI, protecting China from the pollution heaven of FDI.

### 5.2. Promoting Technological Innovation on Energy Saving and Emission reduction

Excessive energy consumption and pollutant emissions are the main sources of environmental inefficiency of China's industry. Technological innovation makes significant contributions to the improvement of the environmental TFP of China's industry. It is necessary to increase research investment to develop environmental technology, energy saving technology, low-carbon technology, and so on.

### 5.3. Improving Energy Consumption Structure

 The government should make policies to promote industrial enterprises to reduce fossil energy consumption including coal and petroleum and improve the proportion of electricity consumption. It is also absolutely essential to vigorously develop clean energy such as nuclear, hydraulic, wind, and solar power.

### 5.4. Establishing Energy and Environment Regulation Policies of Incentive Compatibility

The regulation strategies based on sectors are better than those based on provinces in terms of regulation costs [11]. The emphases of regulation should be shifted to sectors in the short term and take market-oriented instruments of regulation (e.g., prices and taxes) in the long term and especially promote market-oriented reform of electricity and oil industry. The government also should strengthen the pollution control on pollution-intensive industries, such as power production, nonmetallic manufacturing, ferrous metallurgy, paper manufacturing, food processing, and chemical industries which discharge lots of SO_2_ and COD.

## Figures and Tables

**Table 1:** Environmental inefficiency and its components of China's industry.

Category	Industry	Gross industrial output value	Capital stock	Number of employee	Energy consumption	Waste water emission	CO_2_ emission	SO_2_ emission	Solid waste emission	Total value
	Mining	0.195	0.051	0.012	0.119	0.057	0.07	0.076	0.083	0.663
	Manufacturing	0.079	0.068	0.053	0.101	0.060	0.068	0.071	0.065	0.565
VRS	Production and supply of electricity, gas, and water	0.166	0.095	0.013	0.102	0.054	0.057	0.055	0.054	0.596
	Mean value	0.147	0.071	0.026	0.107	0.057	0.065	0.067	0.067	0.608

	Mining	0.249	0.064	0.04	0.123	0.061	0.089	0.081	0.086	0.794
	Manufacturing	0.124	0.088	0.075	0.126	0.067	0.085	0.085	0.081	0.734
CRS	Production and supply of electricity, gas, and water	0.257	0.128	0.023	0.145	0.08	0.085	0.073	0.076	0.867
	Mean value	0.209	0.093	0.046	0.131	0.069	0.086	0.079	0.081	0.798

**Table 2:** Environmental TFP and its components of China's industry.

Industry	LTFP	LPEC	LPTP	LSEC	LTPSC
Mining					
Mining and washing of coal	0.031	−0.1199	0.0975	0.2151	−0.1618
Extraction of petroleum and natural gas	0.0027	−0.2863	0.2561	0.0612	−0.0283
Mining and processing of ferrous metal ores	0.1022	−0.1191	0.0561	0.1826	−0.0175
Mining and processing of nonferrous metal ores	0.1025	−0.0137	0.061	0.0485	0.0067
Mining and processing of nonmetal ores	0.0348	0.0044	0.0535	−0.0242	0.0011
Mean	0.0546	−0.1069	0.1048	0.0966	−0.0399

Manufacturing					
Processing of food from agricultural products	0.0573	−0.0075	0.0264	0.0781	−0.0397
Manufacture of foods	0.0529	−0.0067	0.0586	−0.0158	0.0167
Manufacture of beverages	0.0425	−0.0019	0.04	0.004	0.0005
Manufacture of tobacco	0.0997	0.0294	−0.0415	0.0139	0.0979
Manufacture of textile	0.0227	−0.012	0.0441	−0.0183	0.0090
Manufacture of textile wearing apparel, footware, and caps	0.0247	−0.0317	0.0408	0.0246	−0.009
Manufacture of leather, fur, feather, and related products	0.0089	−0.0212	0.0209	0.0014	0.0078
Processing of timber, manufacture of wood, bamboo, rattan, palm, and straw products	0.0222	−0.0134	0.026	0.0082	0.0014
Manufacture of furniture	0.0870	−0.0173	−0.0830	0.0895	0.0978
Manufacture of paper and paper products	0.0117	−0.0004	0.0502	−0.0963	0.0581
Printing and reproduction of recording media	0.0450	0.0289	−0.0353	0.0168	0.0346
Manufacture of articles for culture, Education, and sport activities	0.0558	0.0034	−0.1198	−0.0533	0.2254
Processing of petroleum, coking, and processing of nuclear fuel	0.0104	−0.0146	0.0444	−0.0487	0.0293
Manufacture of raw chemical materials and chemical products	0.0587	−0.0035	0.0412	0.016	0.0051
Manufacture of medicines	0.0272	−0.0058	0.0182	0.0228	−0.008
Manufacture of chemical fibers	0.0187	0.0025	0.0216	−0.0027	−0.0026
Manufacture of rubber	0.0213	−0.009	0.0265	0.0034	0.0004
Manufacture of plastics	0.0859	−0.0656	0.058	0.054	0.0395
Manufacture of nonmetallic mineral products	0.0755	−0.0094	0.0943	−0.0491	0.0398
Smelting and pressing of ferrous metals	0.0893	0.0093	0.0534	0.0219	0.0047
Smelting and pressing of nonferrous metals	0.0494	−0.0038	0.038	0.0228	−0.0076
Manufacture of metal products	0.0219	−0.0172	0.0287	0.0188	−0.0084
Manufacture of general purpose machinery	0.0783	0.0237	0.0532	−0.0009	0.0023
Manufacture of special purpose machinery	0.0516	0.0006	0.0579	−0.0034	−0.0035
Manufacture of transport equipment	0.0457	0.0035	0.0354	0.0066	0.0002
Manufacture of electrical machinery and equipment	0.0756	−0.0399	0.0954	0.0123	0.0079
Manufacture of communication equipment, computers, and other electronic equipment	0.1757	0.0014	0.0925	0.0534	0.0284
Manufacture of measuring instruments and machinery for cultural activity and office work	0.1463	0.0332	0.0216	0.0900	0.0014
Mean	0.0558	−0.0052	0.0288	0.0096	0.0225

Production and supply of electricity, gas, and water					
Production and supply of electric power and heat power	0.0317	0.0144	0.0145	−0.0574	0.0603
Production and supply of gas	0.0703	0.0101	0.0176	0.0169	0.0257
Production and supply of water	−0.0273	−0.0200	0.0104	−0.0195	0.0018
Mean	0.0249	0.0015	0.0142	−0.0200	0.0293

Total mean	0.0451	−0.0369	0.0493	0.0288	0.0039

**Table 3:** Estimation results of environmental efficiency and environmental TFP^a^.

Variable	Coefficient estimates		
Predictor	Environmental efficiency		Environmental TFP
	VRS	CRS	
Intercept	3.393 (3.334)***	2.283 (2.209)***	2.561 (3.227)***
*X*_1_	−7.234 (−6.789)***	−7.583 (−7.968)***	−8.821 (−10.271)***

*X*_2_	0.16 (2.968)***	0.158 (1.260)	0.172 (3.234)***
*X*_3_	2.747 (0.994)	2.969 (1.227)	3.066 (3.485)***
*X*_4_	15.031 (6.092)***	16.213 (6.256)***	15.615 (7.162)***
*X*_5_	−0.157 (−2.314)**	−0.196 (−2.206)**	0.034 (5.413)***
*R* ^2^ (sigma)	0.064	0.035	0.894
Observations	396	396	360

^a^The standard errors of coefficient estimates are in parentheses. ** and *** denote significance at 5% and 1% levels, respectively.
